# Life Course Approaches to Family Relationships and Health in Later Life: The Long-Lasting Shadow of Childhood Trauma

**DOI:** 10.1093/geroni/igaf122.2106

**Published:** 2025-12-31

**Authors:** Jooyoung Kong, Stephanie Robert, Karen Roberto

## Abstract

Childhood trauma can have lasting effects on individuals’ lives, influencing relationships, health, and well-being. This symposium presents four studies that explore the intersection of childhood trauma, as assessed by Adverse Childhood Experiences (ACEs), family dynamics, and later-life functioning and health through a life course framework. Drawing on longitudinal data from the Midlife in the United States (MIDUS), National Longitudinal Study of Adolescent to Adult Health (Add Health), and Wisconsin Longitudinal Study (WLS), these studies examine how ACEs shape sibling relationships, intergenerational caregiving and support, and social isolation in adulthood. Study 1 investigates the impact of childhood sibling relationships and ACEs on cognitive functioning in late adulthood, emphasizing the role of adult sibling closeness and contact. Study 2 examines the daily experiences of adult-child caregivers, exploring how ACEs influence daily caregiving outcomes and the role of family support and strain. Study 3, grounded in the support bank model, explores how ACEs may affect adult children’s provision of financial or instrumental support when caregiving needs arise in aging parents. Study 4 investigates the association between cumulative ACEs and social isolation in adulthood, examining how family support and strain mitigate or exacerbate these effects. Together, these studies underscore the significance of family relationships across the life course, offering insights into how childhood trauma may influence later-life social functioning and health. This symposium contributes to a deeper understanding of the complex interplay between ACEs, family dynamics, and health, emphasizing the importance of trauma-informed approaches to promoting resilience and well-being across the life course.

